# Clinical decisions on diagnosis and indication for treatment in stereotypical vs. non-stereotypical cases of eating disorders

**DOI:** 10.1186/s12888-026-08407-0

**Published:** 2026-07-17

**Authors:** Sophia Fürtjes, Anne-Christin Luther, Sarah Karg, Katja Beesdo-Baum, Stephanie K. V. Peschel

**Affiliations:** 1 Behavioral Epidemiology, Institute of Clinical Psychology and Psychotherapy, Technische Universität Dresden, Dresden, Germany; 2 Behavioral Epidemiology, Institute of Clinical Psychology and Psychotherapy, TUD Dresden University of Technology, Dresden, Germany

**Keywords:** Eating disorders, Case vignettes, Clinical decision-making, Misjudgement

## Abstract

**Background:**

Prevalence of non-stereotypical Eating Disorders (EDs; e.g., characterized by normal weight, subjective binge eating) is much higher compared with stereotypical EDs (e.g., characterized by under- or overweight, objective binge eating), but treatment rates are much lower. Despite the similar subjective burden, individuals with non-stereotypical symptom presentation are often misperceived and do not receive adequate treatment.

**Methods:**

Using case vignettes, we investigate not only whether non-stereotypical cases of ED are judged differently by a sample of *N* = 308 psychotherapists in training than stereotypical cases. Via a quasi-randomized, controlled design, we also investigate whether a short, educational intervention improves clinical judgement.

**Results:**

Data analyses via hierarchical modelling confirm that non-stereotypical vignettes are perceived as less severe than stereotypical vignettes. They are also less likely to receive an ED-diagnosis, and treatment is less likely to be seen as indicated. The intervention reduced this difference in judgement, leading to a higher perceived problem severity, a higher likelihood of ED-diagnosis, and a higher rating in treatment urgency for non-stereotypical vignettes.

**Conclusions:**

The results draw attention to the problem of misjudgement of non-stereotypical cases of ED. The promising findings regarding the intervention indicate that education of healthcare professionals might contribute to higher likelihood of treatment for individuals with non-stereotypical symptom presentation.

**Clinical trial number:**

Not applicable.

**Supplementary Information:**

The online version contains supplementary material available at 10.1186/s12888-026-08407-0.

## Background

Research has shown that many individuals who meet the diagnostic criteria for an eating disorder (ED) present with characteristics and clinical symptoms which do not meet ED stereotypes. Frequent stereotypes include e.g., the skinny, white, affluent girl (Anorexia nervosa; AN), or the fat, unhappy, incompetent woman (Binge Eating Disorder; BED) [1, 2]. Not only is it concerning that such cliché-based stereotypes are stigmatizing, but also that they are common among mental health professionals [3–5]. Especially weight stigma and internalized thin ideal have been reported to be present health professionals in previous studies [3, 4, 6]. Therefore, individuals with EDs who do not meet the cliché are often overlooked in diagnosis and treatment [5, 7, 8] – even though such cases are highly common [9].

In contrast to such stereotypes as described above, non-stereotypical cases of EDs thus are characterized by e.g., male gender, or non-white ethnicity, but also symptom-related aspects may deviate from ED stereotypes. For example, an individual might not be underweight even though there has been considerable food restriction and weight loss [10]. Another individual might refrain from purging and compensate for binge eating through exercise instead [11], or might not consume objectively large amounts of food despite subjective loss of control during a binge eating episode [12]. These patients nevertheless report comparable or even higher subjective burden [11, 13, 14]. As many studies have shown, quality of life, emotional and social wellbeing are severely impaired in individuals with any form of ED, not only in those whose clinical presentation meets the stereotypes [15–17].

Such cases tend to be more prevalent than stereotypical EDs [9]. An important indicator for this the high prevalence of individuals who do not meet all diagnostic criteria for a specified ED, but instead are diagnosed with EDs in residual diagnostic categories (“atypical” or “other specified/unspecified eating disorders”) because, e.g., their weight or compensatory behaviors do not meet the diagnostic criteria for a specified ED. These cases, which are (a) non-stereotypical in the sense discussed above, and also (b) atypical in regard to the diagnostic category (as opposed to specified diagnoses), show point prevalence rates of up to 10%, compared to 2.8% for AN, 1.5% for BN, or 2.3% for BED [9]. This indicates an association between sociocultural stereotypes (as described above), symptom constellations (i.e., regarding weight or eating behavior), and diagnostic categories (specified/typical vs. unspecified/atypical). These concepts are partially overlapping, e.g., when a patient with extremely restrictive eating has normal weight – which does not meet the sociocultural stereotype, and (in context with other symptoms) often leads to assignment of an atypical/unspecified diagnostic category. In other cases, the overlap might be less pronounced, e.g., when the sociocultural stereotype is not met by a patient with male gender, but the clinical symptoms indicate a specified diagnosis. Sociocultural stereotype, symptom presentation, and diagnostic category are therefore associated with each other, but not synonymous.

The high prevalence of non-stereotypical cases of ED, which overlap with the unspecified/atypical diagnostic category, has been discussed and addressed in the newest version of the International Classification of Diseases (ICD), the ICD-11 [18]. While - as discussed above - patients with non-stereotypical clinical presentations often used to fall into residual diagnostic categories in the ICD-10, they are now allocated to the diagnoses of specified EDs [19]. The current version of the Diagnostic and Statistical Manual of Mental Disorders (DSM-5) [20], on the other hand, still provides more conservative symptom-requirements, such as objectively large amounts of food consumed during a binge eating episode for BED or underweight for AN, to meet the criteria for these specified disorders. Patients who do not meet these symptoms remain in the residual diagnostic category “other specified feeding and eating disorders”.

Previous research suggests that EDs are generally rather poorly recognized by health professionals [5, 7]. Critically, despite the high burden, patients who do not show the stereotypical presentation of EDs are often not recognized by healthcare professionals and therefore often remain untreated. Studies using case vignettes have shown that especially non-stereotypical presentations of EDs are often not recognized by health professionals and are likely to receive different treatment recommendations compared to stereotypical presentations [3, 5, 6, 12, 19, 21]. Similarly, studies using real-life patient data show that individuals with non-stereotypical symptom presentations, such as higher weight or less frequent bingeing, are often not diagnosed correctly [2, 22]. Research investigating treatment rates in EDs also indicates that individuals with non-stereotypical symptoms (e.g., excessive exercise instead of purging, normal weight) are less likely to receive treatment despite the higher prevalence [23, 24].

This raises the question whether interventions such as educational campaigns or including information on non-stereotypical EDs in training programs for mental health professionals might be able to improve diagnostic and treatment of such patients. To our knowledge, in the only previous study investigating this question used a highly comprehensive online program including over 17 h of training to successfully improve the knowledge of health professionals regarding EDs [25, 26]. Whether interventions that are shorter and more resource-efficient might also yield positive effects currently remains unknown.

In summary, non-stereotypical EDs show high prevalence and high subjective burden, but are underdiagnosed and treatment is often suboptimal. Some preliminary evidence suggests that specific information and training could improve clinical decision making in the context of non-stereotypical EDs [19, 26], but research on this matter is scarce. The present study aims to fill this gap. Using case vignettes, we investigate whether non-stereotypical cases of EDs are judged differently by psychotherapists in training than stereotypical cases regarding symptom severity, diagnosis, as well as treatment indication and urgency. We also investigate whether a short, educational intervention improves clinical judgement. The following hypotheses are investigated:

1. Case vignettes with non-stereotypical symptom presentation are judged as less severe, are less often diagnosed, treatment is less likely seen as indicated and perceived as less urgent compared with stereotypical vignettes.
2. In a quasi-randomized, controlled trial, a short intervention educating about the underdiagnosis, undertreatment, and high subjective burden in individuals with non-stereotypical EDs reduces the differences in clinical judgement between stereotypical vs. non-stereotypical vignettes.

## Materials & methods

Study aim, methods, study design, and analyses were preregistered at AsPredicted (https://aspredicted.org/2BM_SWX). Explanations regarding slight deviations from the preregistration will be explained in the analyses section.

### Sample

The study sample consisted of psychotherapists in training, recruited via e-mail through training institutes in Germany. This population was chosen to ensure a homogeneous sample (in contrast to including different groups of mental healthcare professionals), which is currently being trained in clinical decision making. Psychotherapeutic training mandatorily includes assignment of diagnosis according to ICD, as well as treatment indications and contraindications. Recruitment refrained from mentioning EDs to avoid participation bias regarding individuals with a special interest in this subject. Instead, it was more generally stated that the study investigated factors influencing clinical decision-making in the general context of mental disorders. Inclusion criteria were current training to become a psychological psychotherapist according to the German law of 1998, initiation of training at least three months prior to study participation, and German language skills at a native level. Participation was not compensated, but participants could sign up for a raffle of ten 50€ gift cards among participants on a voluntary basis. *N* = 396 individuals started the survey, 310 participants fully completed the survey, which, in line with the pre-registration, was the prerequisite for inclusion within the data analyses. Two participants were excluded from analyses, because, within a textbox providing an opportunity for participant feedback at the end of the survey, one person stated that they had only skimmed instructions and vignettes, and one person stated that they wanted to change their answer in retrospect. Therefore, the final sample size was *N* = 308.

### Measures and materials

The nine vignettes used in the study presented the three EDs (AN, BN, BED) with three clinical presentations each (stereotypical, non-stereotypical, non-pathological). The vignettes were written by two licensed psychotherapists under the consideration of recommendations regarding vignettes for clinical decision making [27] and reviewed by ED experts (clinicians and scientists). The stereotypical vignettes met diagnostic criteria for AN, BN, and BED according to the ICD-10, ICD-11, and DSM-5. The non-stereotypical vignettes described non-stereotypical cases for these disorders according to ICD-11, which also met diagnostic criteria for “atypical” or “other specified” EDs (vignettes depicting AN and BN respectively) or “unspecified” EDs (vignette depicting BED) according to ICD-10 and DSM-5.

The stereotypical and non-stereotypical AN vignettes described the same symptoms and behaviors, the only factor that was manipulated was the bodyweight (underweight vs. normal weight with significant previous weight loss). The non-pathological vignette described fasting during the Christian lent. The stereotypical and non-stereotypical BN vignettes differed only regarding compensatory measures after binge episodes (vomiting vs. excessive exercise). The non-pathological vignette described eating and exercise behaviors of a hobby-cyclist. For the BED vignette, the factor that was manipulated was the amount of food consumed during binge episodes (objectively vs. subjectively large amount). Since the amount of food eaten within subjective vs. objective binge eating episodes is not clearly defined within diagnostic criteria, description of foods consumed within subjective binge eating in the present work was adapted from a previous study [12]. The non-pathological vignette described snacking behavior without loss of control. We decided to focus on these symptom-related aspects of ED stereotypes for our study to enable a clear manipulation with only one aspect differing between stereotypical vs. non-stereotypical vignettes. The complete vignettes can be found in the supplementary material.

#### Vignette ratings

For each vignette, participants rated problem severity on a scale from 1 “not problematic at all” to 10 “very problematic” and the presence of any ED (in accordance with ICD-11) via a dichotomous answer (yes/no). Diagnostic criteria were not provided alongside the vignettes to increase similarity to real-life diagnostic judgements. If presence of any ED was affirmed, the specific diagnosis was selected from the four options AN, BN, BED, and “other”. A dichotomous variable representing a correct diagnosis according to ICD-11 (yes/no) was generated from this answer. Whether or not treatment was considered as indicated was assessed via a dichotomous answer (yes/no). If indication for treatment was affirmed, perceived urgency of treatment was rated on a scale from 1 “not urgent at all” to 10 “very urgent”. Respective items were developed by the study authors.

### Procedure and intervention

The study protocol was evaluated by the ethics committee of the TUD Dresden University of Technology without concerns or objections (EK-156042023). Data were collected via the online survey tool limesurvey.org [28] from 2023/06 to 2023/07. Participants opened the study via a link provided in the recruitment e-mail, read information on the study and on data security, and gave informed consent for participation and data collection. The survey started with questions regarding sociodemographic aspects and the current status of their training. In the first step of the quasi-randomization, participants were then quasi-randomized via alternating allocation to two groups (the two groups did not differ regarding demographic variables; see Table S1 in the supplementary material). The intervention group read a short text with information on the low rates of diagnoses and treatment, as well as high distress in individuals with EDs who show a non-stereotypical clinical presentation (see supplementary material). The control group read a text of similar length informing about the general increase in ED prevalence and the related relevance for healthcare professionals. After the intervention, the second step of the quasi-randomization was employed. Participants were presented with three case vignettes (AN, BN, BED) in quasi-random order by iterating through a list of all possible vignette combinations by group, so that each group was presented with each possible vignette combination. Quasi-randomization was chosen over full randomization to ensure that each of the 72 possible combinations of the three factors intervention, vignette manipulation, and disorder was shown at least three times. The randomization-procedure was programmed on the survey platform. For each participant, one of the vignettes depicted a stereotypical ED, one depicted a non-stereotypical ED, and one described non-pathological eating and exercise behaviors (see details below and also supplementary material). After each vignette, participants provided ratings regarding symptom severity, the presence or absence of any ED-diagnosis (which would also include residual categories) according to ICD-11, the specific diagnosis they would assign if they affirmed presence of any diagnosis, treatment indication, and treatment urgency. With respect to the diagnosis, participants were encouraged to provide the answer they deemed most likely if they were unsure. We chose the ICD, not the DSM, as the frame of reference in the instructions because the ICD is mandatory within the German healthcare system; therefore, therapists need to be familiar with diagnostic criteria according to ICD (not just DSM). Even though the ICD-10 is currently still being used in practice, training already covers ICD-11 – we therefore chose to name the most current version as the frame of reference for diagnosis. These single items used for vignette ratings were created for this study to assess these aspects of clinical judgement in an economic manner, because pre-validated measures for this specific assessment were not available. The development of the items was informed by real-world aspects of clinical decision-making which clinicians are likely to face on a regular basis.

At the end of the survey, participants filled out questionnaires on attitudes towards ED, personal experience with ED, expertise regarding treatment of ED, and knowledge of diagnostic criteria of ED. Afterwards, they were debriefed about the aim of the study and had the option to enter the gift card lottery.

Given the exploratory nature of the present study and for the sake of statistical power, we did not set an a-priori limit to the number of participants we aimed to recruit, but strived for the highest possible number of participants within the pre-defined study period. To ensure that each quasi-randomized combination of intervention vs. control and three out of nine vignettes was answered at least once, a minimum of 72 complete data sets would have sufficed. Under the conservative assumptions of a small effect size (*f* = 0.15), α = 0.05, and β = 0.80, the mixed design requires a sample size of at least *N* = 74 (*n* = 37 per group). The final sample size of *N* = 308 exceeds this number by far, ensuring sufficient quasi-randomization.

### Analyses

Data were analysed using Stata Version SE17.0 [29]. Analyses were conducted on all stereotypical and non-stereotypical vignette data. The non-pathological vignettes served to verify that the intervention did not cause an overshooting effect (i.e., judgement of non-pathological cases as problematic) and were not included in the statistical models. (See *Table **S2* for comparison of intervention vs. control group regarding ratings on the control vignettes; no overshooting effect was found.) The nested data structure with different vignettes (level 1) nested into participants (level 2) requires hierarchical modelling. Hierarchical models with random intercepts and fixed effects for slopes were calculated for perceived problem severity (1–10), assignment of any diagnosis (0/1), indication for treatment (0/1), and urgency of treatment (1–10) as outcomes. In addition, a model with correct diagnosis according to ICD-11 (0/1) as outcome was calculated to explore whether the new criteria, which differ from ICD-10/DSM-5, might already be reflected in diagnostic decisions. Models predicting dichotomous outcomes were logistic models, models predicting continuous outcomes were linear models. To investigate the impact of the vignette-manipulation (stereotypical vs. non-stereotypical, 0/1) on the clinical judgement of the vignettes, this variable was entered as a predictor on level 1 in all models. To investigate the impact of the intervention (control vs. intervention, 0/1), the cross-level interaction manipulation x intervention was included in the models. For the linear models, the assumption of normality of residuals was confirmed. For all models, a likelihood-ratio test confirmed the superiority of the model over an empty model. Contrary to the preregistration, the main effect of intervention was omitted from analyses, because model fit (based on the Bayesian Information Criterion and Akaike Information Criterion) was nearly identical between models with vs. without the additional main effect. We therefore decided to report the more parsimonious models with better interpretability in terms of content. For reasons of transparency, the preregistered models with only the main effect of manipulation excluding the interaction with intervention can be found in the supplementary material, but are not discussed here for reasons of brevity and clarity. All models also included the disorder displayed in the vignette (BED/AN/BN, 0/1/2) as an adjustment variable on level 1. While this deviates from our preregistration, this addition was considered thematically significant due to previously described differences in the probability of correct diagnoses in different EDs [22].

## Results

The sample characteristics can be found in Table 1, the descriptive statistics of the outcome variables are displayed in Table 2. Stereotypical vignettes were rated higher in problem severity than non-stereotypical vignettes (t = 10.57, *p* < .001) and more often received an ED-diagnosis (χ2 = 51.70, *p* < .001). They were also more likely to be assigned the correct diagnosis according to ICD-11 (χ2 = 155.45, *p* < .001). For stereotypical vignettes, treatment was also more often seen as indicated (χ2 = 10.28, *p* = .001) and rated as more urgent (t = 8.07, *p* < .001) than for non-stereotypical vignettes.

**Table 1:** Sample characteristics [*N* = 308]

Age [*M* ± *SD*]	31.09 ± 5.57
Sex [*n* (%)]	
Female	272 (88.3)
Male	33 (10.7)
Other	3 (1.0)
*Training for* [*n* (%)]	
Psychological psychotherapist	188 (61.0)
Child and adolescent psychotherapist	102 (33.1)
Both	18 (5.8)
*School of training* [*n* (%)]^†^	
CBT	199 (64.6)
PDT	74 (24.0)
Psychoanalysis	32 (10.4)
Systemic Therapy	34 (11.0)

Notes. CBT = Cognitive-behavioral therapy; PDT = Psychodynamic therapy. †Multiple selection possible

**Table 2:** Descriptive statistics of the five outcome variables

	Stereotypical [*N* = 308]			Non-stereotypical [*N* = 308]		
	AN [*n* = 108]	BN [*n* = 112]	BED [*n* = 88]	AN [*n* = 99]	BN [*n* = 97]	BED [*n* = 112]
Problem severity [*M* ± *SD*]	9.39 ± .93	8.47 ± 1.28	8.34 ± 1.45	8.24 ± 1.29	7.60 ± 1.51	6.30 ± 2.34
ED present [*n* (%)]	105 (97.2)	107 (95.5)	80 (90.9)	81 (81.8)	81 (83.5)	65 (58.0)
Correct ICD-11 diagnosis [*n* (%)]	103 (95.4)	90 (80.4)	77 (87.5)	44 (44.4)	32 (33.0)	45 (40.2)
Treatment indicated [*n* (%)]	105 (97.2)	111 (99.1)	87 (98.9)	94 (95.0)	96 (99.0)	97 (86.6)
Treatment urgency [*M* ± *SD*]^†^	9.16 ± 1.15	7.92 ± 1.53	7.67 ± 1.68	7.50 ± 1.78	7.14 ± 1.83	6.80 ± 1.79

Notes. AN = Anorexia nervosa; BN = Bulimia nervosa; BED = Binge eating disorder. ED = eating disorder. Problem severity and treatment urgency were rated on a scale from 1 to 10. The other variables are dichotomous (yes/no). Percentages refer to the n of the column. †Only rated if treatment was considered as indicated

The hierarchical analyses revealed a significant main effect of manipulation (stereotypical vs. non-stereotypical) on all outcomes (see Table 3 and 4), confirming that stereotypical vignettes were perceived as more problematic than non-stereotypical vignettes, were diagnosed more often, were more likely to be perceived as in need for treatment, and treatment was considered to be more urgent. The odds rations (dichotomous outcomes) and betas (continuous outcomes on a scale from 1 to 10) indicate small effect sizes, with the strongest effects for treatment indication (OR = 0.15, SE = 0.11, *p* = .013) and perceived problem severity (b = -1.42, SE = 0.16, *p* < .001).

**Table 3:** Results of the hierarchical logistic models predicting vignette ratings - dichotomous outcomes

	ED present			Correct ICD-11 diagnosis			Treatment indicated		
	OR [SE]	*p*	95% CI	OR [SE]	*p*	95% CI	OR [SE]	*p*	95% CI
*Level 1: vignette*									
Manipulation:									
Non-stereotypical	.09 [.04]	< .001	[.04; .22]	.07 [.02]	< .001	[.03; .14]	.15 [.11]	0.013	[.03; .67]
Disorder:									
AN	3.47 [1.02]	< .001	[1.95; 6.19]	1.48 [.37]	0.114	[.91; 2.41]	2.05 [.92]	0.112	[.85; 4.96]
BN	3.38 [1.00]	< .001	[1.90; 6.03]	.64 [.16]	0.077	[.40; 1.05]	8.40 [6.39]	0.005	[1.89; 37.30]
*Cross-level interaction*									
Intervention x stereotypical	.69 [.36]	0.477	[.25; 1.92]	1.07 [.38]	0.858	[.53; 2.13]	.61 [.56]	0.59	[.10; 3.72]
Intervention x non-stereotypical	2.00 [.55]	0.012	[1.16; 3.43]	1.57 [.39]	0.067	[.97; 2.54]	1.71 [.81]	0.257	[.68; 4.30]

Notes. Outcomes were dichotomous (yes/no). OR = odds ratio.  ED = eating disorder. Stereotypical served as base category for the effect of manipulation. AN = Anorexia nervosa; BN = Bulimia nervosa; binge eating disorder served as base for the effect of disorder

**Table 4:** Results of the hierarchical linear models predicting vignette ratings - continuous outcomes

	Problem severity			Treatment urgency^†^		
	b [SE]	*p*	95% CI	b [SE]	*p*	95% CI
*Level 1: vignette*						
Manipulation:						
Non-stereotypical	**-1.42 [.16]**	**< .001**	**[-1.73; -1.11]**	**-1.36 [.16]**	**< .001**	**[-1.66; -1.05]**
Disorder:						
AN	**1.51 [.14]**	**< .001**	**[1.23; 1.80]**	**1.09 [.19]**	**< .001**	**[.80; 1.38]**
BN	**.77 [.14]**	**< .001**	**[.49; 1.05]**	**.33 [.15]**	**0.023**	**[.05; .62]**
*Cross-level interaction*						
Intervention x stereotypical	.33 [.18]	0.064	[-.02; .67]	.21 [.19]	0.278	[-.17; .59]
Intervention x non-stereotypical	**.48 [.18]**	**0.007**	**[.13; .82]**	**.64 [.19]**	**0.001**	**[.27; 1.02]**

Notes. Outcomes were rated on a scale from 1 to 10. Stereotypical served as base category for the effect of manipulation. AN = Anorexia nervosa; BN = Bulimia nervosa; binge eating disorder served as base for the effect of disorder. ^†^Only rated if treatment was considered indicated

**Fig. 1:**
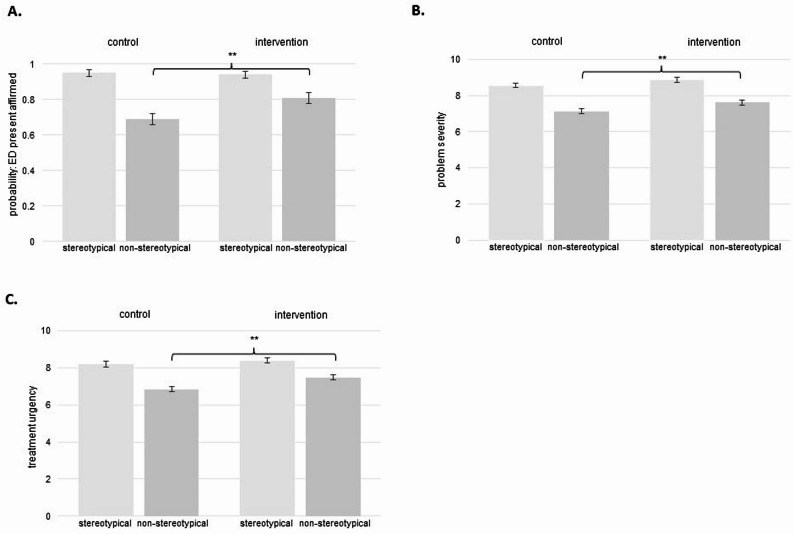
Interaction between vignette-manipulation (stereoptypical vs. non-stereoptypical) and intervention on clinical judgement. N = 308; ** = significant at α ≤ 0.01. (**A**) Dichotomous outcome ED present (yes/no). (**B**) Continuous outcome problem severity, rated on a scale from 1 to 10. (**C**) Continuous outcome treatment urgency, rated on a scale from 1 to 10

The significant interaction of the vignette-manipulation with the intervention showed that the difference between ratings on stereotypical vs. non-stereotypical vignettes was reduced in the intervention group for the outcomes problem severity, any diagnosis, and treatment urgency (see Fig. 1; see Table S7 + S8 in the supplementary for the predicted marginal effects). In the intervention group, non-stereotypical vignettes were perceived as more problematic, more often received a diagnosis, and treatment was perceived as more urgent than in the control group. Again, odds ratio and betas indicate that effect size is small. Even though the difference in ratings between stereotypical vs. non-stereotypical vignettes was reduced in the intervention group, it did not disappear completely. There was no significant effect of the intervention regarding the differences between stereotypical and non-stereotypical vignettes for treatment indication and correct diagnosis according to ICD-11.

Perceived problem severity, likelihood of any diagnosis, and perceived urgency of treatment were higher for AN and BN vignettes compared with BED (see Tables S3 + S4 in the supplementary material). Perceived problem severity and urgency of treatment were higher for AN vignettes compared with BN.

## Discussion

Our analyses revealed that in comparison with stereotypical cases, ED vignettes with non-stereotypical symptom presentation are perceived as less severe, are often misdiagnosed, and treatment is less likely to be considered indicated and urgent. The present findings confirm previous reports of misperception, misdiagnosis, and suboptimal treatment in non-stereotypical EDs [2, 3, 12, 19, 21, 22]. It should be noted that even for non-stereotypical cases, a relatively high percentage of participants assigned a diagnosis to the vignette case (83.5% for BN, 81.8% for AN, but only 58.0% for BED) and saw an indication for treatment (99.0% BN, 95.0% AN, 86.6% BED). While these numbers are encouraging considering that arguably recognizing need for treatment would probably be most important aspect of clinical judgement in a real-life healthcare setting, it is nevertheless problematic that individuals who do not represent the ED stereotypes receive less recognition than cases who do – even when effect sizes are small. While, e.g., a difference of 15.4% (AN) or 12.0% (BN) in assigned diagnosis might not seem like much on paper, but for those individuals who fall under the radar such systematic differences still matter. Even small differences in more nuanced aspects of clinical judgement, such as perceived problem severity and treatment urgency, can contribute to problematic undertreatment in individuals who are being taken less seriously because they do not fit the ED stereotypes.

Previous research involving ED-case vignettes has often focused on general practitioners [7, 30], counsellors [5], or even healthcare professionals completely outside the field of mental health [8]. Furthermore, samples were generally heterogeneous. For example, clinical psychologists mixed with fitness instructors participated in Worsfold & Sheffield (2018) [31]; general practitioners, psychiatrists, nurses, and dieticians represented the sample of Cain et al. (2017) [32]. Such methodological aspects limit the interpretability of reported results. Our findings confirm and underline the severity of the underdiagnosis of non-stereotypical EDs even in professional psychologists who are in training to become psychotherapists - i.e., a group of healthcare professionals whose job includes diagnosis and treatment of EDs. Especially considering the high prevalence and high burden of non-stereotypical EDs, our findings indicate that awareness of and level of information regarding non-stereotypical EDs in mental health professionals need to be improved.

It is therefore promising that we found a short, economic educational intervention to be associated with improvement in different aspects of clinical judgement in the short-term context of the vignette-based study. Perception of problem severity and treatment urgency was significantly increased in participants who received the brief intervention. While the difference in judgement of stereotypical vs. non-stereotypical cases remained present in both groups, the intervention was able to significantly attenuate this effect. Previously, an extensive online training program with over 17 h of training has been shown to improve health professionals’ knowledge regarding EDs [25]. The present study suggests that even much shorter, targeted interventions may also carry the potential to yield beneficial effects at least regarding clinical judgement. Future research should further investigate the effects of such interventions over a longer period of time and in the context of real-life decision-making. If the effect can be supported by future research, including such targeted education in the context of training future mental health professionals should be taken into consideration. Our findings show a bias in different aspects of clinical decision making (perceived problem severity, treatment indication etc.) regarding non-stereotypical cases of ED in therapists in training – and the potential of intervening at this very early stage of working with clients. If training included such interventions early in, biases might be addressed and reduced early in the career of mental health professionals. Additionally, testing the effectiveness of such interventions in healthcare professionals outside the mental health sector who nevertheless provide care for individuals with EDs, such as general practitioners, could also present a worthwhile goal for future studies. If studies were to show promising results, short information campaigns for other healthcare professionals such as general practitioners might also contribute to better healthcare service for individuals with ED who present in a non-stereotypical manner.

Our findings should be considered against the backdrop of several limitations. First, our sample consisted of psychotherapists in training, therefore results cannot be generalized to other groups of healthcare professionals such as psychiatrists, general practitioners, etc. Future studies are needed to investigate whether our findings can be replicated in such samples. Especially clinicians specialized in ED treatment are likely to differ from our sample – which is something that future research should consider investigating. Second, it is important to consider that (even though vignette manipulation was carried out carefully and vignettes were reviewed by clinical experts) manipulation might not be equal regarding diagnostic ambiguity for all vignettes. Also, certain aspects, such as e.g., religious fasting as non-pathological restrictive eating, might be perceived differently by raters of differing sociocultural backgrounds. Replication of our findings in future studies using a larger variety of vignettes and/or adjusting for diagnostic ambiguity is needed to provide more certainty. Future studies should also explore manipulating different aspects of non-stereotypical presentations, since the findings regarding symptom-related aspects might not generalize to other facets of ED stereotypes (e.g., gender, ethnicity). It should also be considered that the complex study-design (intervention vs. control x stereotypical vs. non-stereotypical vs. non-pathological x AN vs. BN vs. BED) only allowed for quasi-randomization, not full randomization, which limits internal validity of the results. The single-item measures for vignette ratings present a further limitation, since – even though it is common practice to rely on custom items in vignette studies due to the specificity of the assessments – they are not pre-evaluated regarding psychometric quality. Due to the vignette-based study-design, we can also not draw conclusions about clinical decision-making in the actual workplace environment with real-life patients, and consider specifics of patients e.g. limited insight which might interact with clinical decision making. While well-designed vignette-studies provide reliable information on decision-making processes and external validity is often high [27, 33], our findings still need to be extended into studies investigating actual decisions of clinicians in their workplace. Even though it is encouraging to see the significant effect of the short educational intervention in our data, we do not know whether this effect persists over a longer period of time. Especially considering the very brief nature of the intervention, the potentially only short duration of effects is an important limitation to our findings. Longitudinal studies are needed to investigate the long-term utility of such interventions. Additionally, the possibility that the intervention effect might also be influenced by social desirability rather than increased sensitivity should be taken into consideration. Lastly, it should be noted that even though they were currently in training where the current “state of the art” of diagnostics is taught, the majority of participants reported that they were not yet intimately familiar with the ICD-11 criteria for EDs, which is likely to impact the outcome “correct diagnosis according to ICD-11”. A (comparatively) low rate of accuracy regarding ICD-11 diagnosis is most likely not due to clinical failure, but to lack of familiarity with the newest diagnostic standard. Diagnostic decisions regarding the specific diagnosis might change when the relatively new ICD-11 criteria become more established. However, since all vignettes fulfilled criteria for an ED diagnosis, whether according to the ICD-10/DSM-5 (where the diagnoses for non-stereotypical vignettes would fall into the atypical or residual categories), or according to the ICD-11 (where they would classify as AN/BN/BED), we deem it unlikely that our general findings regarding the different aspects of clinical decision making such as perceived problem severity or treatment indication, or the effect of the educational intervention, are impacted by this matter.

## Conclusion

In conclusion and in line with previous research, our study draws attention to the problem that individuals with ED who do not represent the stereotype of ED patients are often misdiagnosed and less likely to be seen as in need for treatment. Short educational interventions could potentially be a useful, resource-efficient tool to increase awareness and reduce the treatment-gap.

## Supplementary Information

Below is the link to the electronic supplementary material.

Supplementary Material 1

## Data Availability

All material (case vignettes, intervention) is published alongside the manuscript as supplementary material. The dataset is available from the corresponding author upon request.

## Abbreviations

AN: Anorexia nervosa
BED: Binge eating disorder
BN: Bulimia nervosa
DSM: Diagnostic and Statistical Manual of Mental Disorders
ED: Eating disorders
ICD: International Classification of Diseases

## References

[1] Brelet L, Flaudias V, Désert M, Guillaume S, Llorca P-M, Boirie Y. Stigmatization toward people with anorexia nervosa, bulimia nervosa, and binge eating disorder: A scoping review. Nutrients. 2021;13:2834. 10.3390/nu13082834. 34444994 10.3390/nu13082834 PMC8400545

[2] Sonneville KR, Lipson SK. Disparities in eating disorder diagnosis and treatment according to weight status, race/ethnicity, socioeconomic background, and sex among college students. Int J Eat Disord. 2018;51:518–26. 10.1002/eat.22846. 29500865 10.1002/eat.22846

[3] Veillette LAS, Serrano JM, Brochu PM. What’s weight got to do with it? Mental health trainees’ perceptions of a client with anorexia nervosa symptoms. Front Psychol. 2018;9:5274. 10.3389/fpsyg.2018.02574. 10.3389/fpsyg.2018.02574 PMC6304369 30618990

[4] Puhl RM, Latner JD, King KM, Luedicke J. Weight bias among professionals treating eating disorders: Attitudes about treatment and perceived patient outcomes. Int J Eat Disord. 2014;47:65–75. 10.1002/eat.22186. 24038385 10.1002/eat.22186

[5] McNicholas F, O’Connor C, O’Hara L, McNamara N. Stigma and treatment of eating disorders in Ireland: Healthcare professionals’ knowledge and attitudes. Ir J Psychol Med. 2016;33:21–31. 10.1017/ipm.2015.24. 30115174 10.1017/ipm.2015.24

[6] Worsfold KA, Sheffield JK. Practitioner eating disorder detection: The influence of health mindset, thin-ideal internalization, orthorexia and gender role. Early Interv Psychiatry. 2021;15:296–305. 10.1111/eip.12940. 32196980 10.1111/eip.12940

[7] Currin L, Schmidt U, Waller G. Variables that influence diagnosis and treatment of the eating disorders within primary care settings: A vignette study. Int J Eat Disord. 2007;40:257–62. 10.1002/eat.20355. 17167756 10.1002/eat.20355

[8] Garcia Moreno N, Walker DC, Gullo N, O’Dea CJ. Weight Stigma’s Effects on Misdiagnosis of Eating Disorders Among Laypeople and Healthcare Professionals. Int J Eat Disord. 2025;58:690–702. 10.1002/eat.24374. 39803860 10.1002/eat.24374

[9] Galmiche M, Déchelotte P, Lambert G, Tavolacci MP. Prevalence of eating disorders over the 2000–2018 period: a systematic literature review. Am J od Clin Nutr. 2019;1402–13. 10.1093/ajcn/nqy342. 10.1093/ajcn/nqy342 31051507

[10] Walsh BT, Hagan KE, Lockwood C. A systematic review comparing atypical anorexia nervosa and anorexia nervosa. Int J Eat Disord. 2023;56:798–820. 10.1002/eat.23856. 36508318 10.1002/eat.23856

[11] Jordan J, McIntosh VVW, Carter JD, Rowe S, Taylor K, Frampton CMA, et al. Bulimia nervosa-nonpurging subtype: Closer to the bulimia nervosa-purging subtype or to binge eating disorder? Int J Eat Disord. 2014;47:231–8. 10.1002/eat.22218. 24282157 10.1002/eat.22218

[12] Berner LA, Sysko R, Rebello TJ, Roberto CA, Pike KM. Patient descriptions of loss of control and eating episode size interact to influence expert diagnosis of ICD-11 binge-eating disorder. J Eat Disord. 2020;8. 10.1186/s40337-020-00342-z. 10.1186/s40337-020-00342-z PMC7682053 33292557

[13] Palavras MA, Morgan CM, Borges FMB, Claudino AM, Hay PJ. An investigation of objective and subjective types of binge eating episodes in a clinical sample of people with co-morbid obesity. J Eat Disord. 2013;1:26. 10.1186/2050-2974-1-26. 24999405 10.1186/2050-2974-1-26 PMC4081732

[14] Pauls A, Dimitropoulos G, Marcoux-Louie G, Singh M, Patten SB. Psychological characteristics and childhood adversity of adolescents with atypical anorexia nervosa versus anorexia nervosa. Eat Disord. 2022;30:210–22. 10.1080/10640266.2020.1836907. 33103622 10.1080/10640266.2020.1836907

[15] de Vos JA, Radstaak M, Bohlmeijer ET, Westerhof GJ. Having an eating disorder and still being able to flourish? Examination of pathological symptoms and well-being as two continua of mental health in a clinical sample. Front Psychol. 2018;9:NOV2145. 10.3389/fpsyg.2018.02145. 10.3389/fpsyg.2018.02145 PMC6249270 30498463

[16] Van Hoeken D, Hoek HW. Review of the burden of eating disorders: mortality, disability, costs, quality of life, and family burden. Curr Opin Psychiatry. 2020;33:521–7. 10.1097/YCO.0000000000000641. 32796186 10.1097/YCO.0000000000000641 PMC7575017

[17] Hart LM, Gordon AR, Sarda V, Calzo JP, Sonneville KR, Samnaliev M, et al. The association of disordered eating with health-related quality of life in U.S. young adults and effect modification by gender. Qual Life Res. 2020;29:1203–15. 10.1007/s11136-019-02396-2. 31970623 10.1007/s11136-019-02396-2

[18] World Health Organization. ICD-11: International classification of diseases (11th revision). 2022.

[19] Claudino AM, Pike KM, Hay P, Keeley JW, Evans SC, Rebello TJ, et al. The classification of feeding and eating disorders in the ICD-11: Results of a field study comparing proposed ICD-11 guidelines with existing ICD-10 guidelines. BMC Med. 2019;17:93. 10.1186/s12916-019-1327-4. 31084617 10.1186/s12916-019-1327-4 PMC6515596

[20] American psychiatric association. Diagnostic and statistical manual of mental disorders (DSM-5). 5th edition. American psychiatric publishing; 2013.

[21] Silbiger K. Mental health providers’ perceptions of restrictive eating disorders: Relationship with client body weight. Int J Eat Disord. 2024;57:916–23. 10.1002/eat.24154. 38291927 10.1002/eat.24154

[22] Silén Y, Sipilä PN, Raevuori A, Mustelin L, Marttunen M, Kaprio J, et al. Detection, treatment, and course of eating disorders in Finland: A population-based study of adolescent and young adult females and males. Eur Eat Disorders Rev. 2021;29:720–32. 10.1002/erv.2838. 10.1002/erv.2838 PMC8349843 34008267

[23] Trompeter N, Bussey K, Forbes MK, Mond J, Hay P, Basten C, et al. Differences between Australian adolescents with eating disorder symptoms who are in treatment or not in treatment for an eating disorder. Early Interv Psychiatry. 2021;15:882–8. 10.1111/eip.13027. 32881352 10.1111/eip.13027

[24] Harrop EN, Mensinger JL, Moore M, Lindhorst T. Restrictive eating disorders in higher weight persons: A systematic review of atypical anorexia nervosa prevalence and consecutive admission literature. Int J Eat Disord. 2021;54:1328–57. 10.1002/eat.23519. 33864277 10.1002/eat.23519 PMC9035356

[25] Maguire S, Li A, Cunich M, Maloney D. Evaluating the effectiveness of an evidence-based online training program for health professionals in eating disorders. J Eat Disord. 2019;7:14. 10.1186/s40337-019-0243-5. 31110761 10.1186/s40337-019-0243-5 PMC6513519

[26] Brownlow RS, Maguire S, O’Dell A, Dias-da-Costa C, Touyz S, Russell J. Evaluation of an online training program in eating disorders for health professionals in Australia. J Eat Disord. 2015;3. 10.1186/s40337-015-0078-7. 10.1186/s40337-015-0078-7 PMC4636783 26550477

[27] Evans SC, Roberts MC, Keeley JW, Blossom JB, Amaro CM, Garcia AM, et al. Vignette methodologies for studying clinicians’ decision-making: Validity, utility, and application in ICD-11 field studies. Int J Clin Health Psychol. 2015;15:160–70. 10.1016/j.ijchp.2014.12.001. 30487833 10.1016/j.ijchp.2014.12.001 PMC6224682

[28] Limesurvey GmbH. LimeSurvey: an open source survey tool. 2023.

[29] StataCorp. Stata statistical software. 2019.

[30] Green H, Johnston DClinPsy OB, Cabrini SB, Fornai GP, Trainee Tony Kendrick FRCGP FRCPsych GB. General practitioner attitudes towards referral of eating-disordered patients: a vignette study based on the theory of planned behaviour Clinical Psychologist in Training. Radcliffe Publishing; 2008. PMC2777584 22477872

[31] Worsfold KA, Sheffield JK. Eating disorder mental health literacy: What do psychologists, naturopaths, and fitness instructors know? Eat Disord. 2018;26:229–47. 10.1080/10640266.2017.1397420. 29173080 10.1080/10640266.2017.1397420

[32] Cain B, Buck K, Fuller-Tyszkiewicz M, Krug I. Australian healthcare professionals’ knowledge of and attitudes toward binge eating disorder. Front Psychol. 2017. 10.3389/fpsyg.2017.01291. 8 AUG. 28824484 10.3389/fpsyg.2017.01291 PMC5545598

[33] Steiner PM, Atzmüller C, Su D. Designing valid and reliable vignette experiments for survey research: a case study on the fair gender income gap. 2016.

