# Supplementary material for: Clinical decisions on diagnosis and indication for treatment in stereotypical vs. non-stereotypical cases of eating disorders
Source: BMC Psychiatry. 2026 Jul 17;26:540. doi: 10.1186/s12888-026-08407-0 (PMC13377787; doi:10.1186/s12888-026-08407-0)
Supplement: Supplementary file 1 — Supplementary Material 1 [file 12888_2026_8407_MOESM1_ESM.docx]

| *Table S1*. Sample characteristics reported by group | | | |
| --- | --- | --- | --- |
|  | **intervention  (*n* = 151)** | **control (*n* = 157)** | **group comparison** |
| age [*M* ± *SD*]  *sex* [*n* (%)]  female  male  other  *training for* [*n* (%)]  psychological psychotherapist  child and adolescent psychotherapist  both  *school of training* [*n* (%)]^†^  CBT  PDT  Psychoanalysis  Systemic Therapy  *length of training* [*n* (%)]  < 1 year  1 to max. 2 years  > 2 to max. 4 years  > 4 years  *work experience in facility specialized in ED* [*n* (%)]  yes  no | 31.31 ± 5.31  138 (91.4)  13 (8.6)  0 (0.0)  86 (57.0)  57 (37.8)  8 (5.3)  100 (66.2)  36 (23.8)  18 (11.9)  15 (9.9)  23 (15.2)  44 (29.1)  57 (27.8)  27 (17.9)  127 (84.1)  24 (15.9) | 30.88 ± 5.82  134 (85.4)  20 (12.7)  3 (1.9)  102 (65.0)  45 (28.7)  10 (6.37)  99 (63.1)  38 (24.2)  14 (8.9)  19 (12.1)  39 (24.8)  40 (25.5)  55 (35.0)  23 (14.7)  129 (82.2)  28 (17.8) | *t* = -.068, *p* = .496  χ² = 4.43, *p* = .109  χ² = 2.88, *p* = .237  χ² = .34, *p* = .561  χ² = .01, *p* = .941  χ² = .75, *p* = .388  χ² = .37, *p* = .544  χ² = 4.56, *p* = .207  χ² = .21, *p* = .650 |
| *Notes.* CBT = cognitive-behavioral therapy; PDT = psychodynamic therapy. ^†^ multiple selection possible. ED = eating disorder. | | | |

| *Table S2*. Descriptive statistics for non-pathological vignettes, reported by group | | | |
| --- | --- | --- | --- |
|  | **intervention  (*n* = 151)** | **control (*n* = 157)** | **group comparison** |
| problem severity [*M* ± *SD*]  ED present [*n* (%)]  treatment indicated [*n* (%)] | 2.41 ± .15  4 (2.7)  10 (6.6) | 2.32 ± .13  3 (1.9)  8 (5.1) | *t* = -.50, *p* = .614  χ² = .19, *p* = .664  χ² = .33, *p* = .568 |
| *Notes*. AN = Anorexia nervosa; BN = Bulimia nervosa; BED = binge eating disorder. ED = eating disorder. Problem severity was rated on a scale from 1 to 10. The other variables are dichotomous (yes/no). Percentages refer to the *n* of the column. | | | |

| *Table S3*. Results of the hierarchical logistic models predicting vignette ratings independent of intervention | | | | | | | | | | |
| --- | --- | --- | --- | --- | --- | --- | --- | --- | --- | --- |
|  | **ED present** | | | **correct ICD-11 diagnosis** | | | **treatment indicated** | | | |
|  | ***OR* [*SE*]** | ***p*** | **95 % CI** | ***OR* [*SE*]** | ***p*** | **95 % CI** | ***OR* [*SE*]** | ***p*** | **95 % CI** |  |
| *Level 1: vignette*  manipulation:  non-stereotypical  disorder:  AN  BN | **.16 [.05]**  **3.28 [.96]**  **3.24 [.94** | **< .001**  **< .001**  **< .001** | **[.09; .28]**  **[1.85; 5.80]**  **[1.83; 5.73]** | **.08 [.02]**  1.45 [.36]  .64 [.16] | **< .001**  .131  .071 | **[.05; .14]**  [.89; 2.36]  [.39; 1.04] | **.25 [.12]**  1.99 [.89]  **8.21 [.6.23]** | **.006**  .127  **.006** | **[.09; .66]**  [.82; 4.79]  **[1.85; 36.37]** |  |
| *Notes*. Outcomes were dichotomous (yes/no). ED = eating disorder. Stereotypical served as base category for the effect of manipulation. AN = Anorexia nervosa; BN = Bulimia nervosa; binge eating disorder served as base for the direct effect of disorder. | | | | | | | | | | |

| *Table S4*. Results of the hierarchical linear models predicting vignette ratings independent of intervention | | | | | | |
| --- | --- | --- | --- | --- | --- | --- |
|  | **problem severity** | | | **treatment urgency**^†^ | | |
|  | ***b* [*SE*]** | ***p*** | **95 % CI** | ***b* [*SE*]** | ***p*** | **95 % CI** |
| *Level 1: vignette*  manipulation:  non-stereotypical  disorder:  AN  BN | **-1.35 [.11]**  **1.51 [.15]**  **.77 [.14]** | **< .001**  **< .001**  **< .001** | **[-1.57; -1.13]**  **[1.22; 1.79]**  **[.48; 1.05]** | **-1.14 [.11]**  **1.09 [.15]**  **.32 [.15]** | **< .001**  **< .001**  **.028** | **[-1.36; -.92]**  **[.79; 1.37]**  **[.04; .61]** |
| *Notes*. Outcomes were rated on a scale from 1 to 10. Stereotypical served as base category for the effect of manipulation. AN = Anorexia nervosa; BN = Bulimia nervosa; binge eating disorder served as base for the direct effect of disorder. ^†^only rated if treatment was considered indicated. | | | | | | |

| *Table S5*. Contrast of effects of AN vs. BN vs. BED as estimated in the main analyses of the manuscript | | | | | | | | | | |
| --- | --- | --- | --- | --- | --- | --- | --- | --- | --- | --- |
|  | **ED present** | | | **correct ICD-11 diagnosis** | | | **treatment indicated** | | | |
|  | ***c* [*SE*]** | ***p*** | **95 % CI** | ***c* [*SE*]** | ***p*** | **95 % CI** | ***c* [*SE*]** | ***p*** | **95 % CI** |  |
| AN vs. BED  BN vs. BED BN vs. AN | **1.24 [.30]**  **1.22 [.29]**  -.03 [.34] | **< .001**  **< .001**  .939 | **[.67; 1.82]**  **[.64; 1.80]**  [-.69; .64] | .39 [.25]  -.44 [.25]  **-.83 [.26]** | .114  .077  **.001** | [-.09; .88]  [-.93; .05]  **[-1.34; -.32]** | .72 [.45]  **2.13 [.76]**  1.41 [.80] | .112  **.005**  .078 | [-.17; 1.60]  **[.64; 3.62]**  [-.16; 2.98] |  |
| *Notes*. Outcomes were dichotomous (yes/no). ED = eating disorder. AN = Anorexia nervosa; BN = Bulimia nervosa; BED = binge eating disorder. | | | | | | | | | | |

| *Table S6*. Contrast of effects of AN vs. BN vs. BED as estimated in the main analyses of the manuscript | | | | | | |
| --- | --- | --- | --- | --- | --- | --- |
|  | **problem severity** | | | **treatment urgency**^a^ | | |
|  | ***c* [*SE*]** | ***p*** | **95 % CI** | ***c* [*SE*]** | ***p*** | **95 % CI** |
| AN vs. BED  BN vs. BED BN vs. AN | **1.51 [.14]**  **.77 [.14]**  **-.74 [.14]** | **< .001**  **< .001**  **< .001** | **[1.23; 1.80]**  **[.49; 1.05]**  **[-1.02; -.46]** | **1.08 [.15]**  **.33 [.15]**  **-.75 [.14]** | **< .001**  **.023**  **< .001** | **[.79; 1.37]**  **[.05; .62]**  **[-1.03; -.47]** |
| *Notes*. Outcomes were rated on a scale from 1 to 10. AN = Anorexia nervosa; BN = Bulimia nervosa; BED = binge eating disorder. ^a^ only rated if treatment was considered indicated. | | | | | | |

| *Table S7*. Predicted margins for the interaction effect of intervention x manipulation as estimated in the main analyses of the manuscript | | | | | | |
| --- | --- | --- | --- | --- | --- | --- |
|  | **ED present** | | **correct ICD-11 diagnosis** | | **treatment indicated** | |
|  | ***PMM* [*SE*]** | **95 % CI** | ***PMM* [*SE*]** | **95 % CI** | ***PMM* [*SE*]** | **95 % CI** |
| stereotypical x control  stereotypical x intervention  non-stereotypical x control  non-stereotypical x intervention | .95 [.029  .94 [.02]  .69 [.04]  .81 [.03] | [.92; .99]  [.90; .98]  [.62; .76]  [75; .87] | .87 [.03]  .88 [.03]  .34 [.04]  .44 [.04] | [.82; .93]  [.83; .93]  [.27; .42]  [.37; .52] | .99[.01]  .98 [.01]  .92 [.02]  .95 [.02] | [.97; 1.00]  [.95; 1.00]  [.88; .96]  [.92; .98] |
| *Notes*. Outcomes were dichotomous (yes/no). PMM = predicted marginal mean. ED = eating disorder. AN = Anorexia nervosa; BN = Bulimia nervosa; BED = binge eating disorder. | | | | | | |

| *Table S8*. Predicted margins for the interaction effect of intervention x manipulation as estimated in the main analyses of the manuscript | | | | |
| --- | --- | --- | --- | --- |
|  | **problem severity** | | **treatment urgency**^a^ | |
|  | ***PMM* [*SE*]** | **95 % CI** | ***PMM* [*SE*]** | **95 % CI** |
| stereotypical x control  stereotypical x intervention  non-stereotypical x control  non-stereotypical x intervention | 8.56 [.12]  8.88 [.13]  7.13 [.12]  7.61 [.13] | [8.31; 8.80]  [8.64; 9.13]  [6.89; 7.38]  [7.37; 7.86[ | 8.20 [.14]  8.40 [.14]  6.84 [.14]  7.48 [.14] | [7.93; 8.46]  [8.14; 8.67]  [6.57; 7.10]  [7.21; 7.75] |
| *Notes*. Outcomes were rated on a scale from 1 to 10. AN = Anorexia nervosa; BN = Bulimia nervosa; BED = binge eating disorder. ^a^ only rated if treatment was considered indicated. | | | | |

**Vignettes (the original vignettes used within the study were in German; an English translation is provided below)**

*BED - stereotypical:* Ms. G., 27 years old, reports that she has “big problems with food” and “just doesn’t know what to do anymore.” Even at primary school age, she had a higher weight than most of her peers, which is why her mother “put her on a diet early on”. She currently weighs 81 kg and is 170 cm tall (BMI 28 kg/m²). Her eating habits have been “completely out of control” for some time now: for about 1.5 years now, she has been “shoving down an extremely large amount of food at once” about every two to three days, even though she is not hungry at all. In such situations, for example, she consumes six slices of toast with butter and salami, two 100 g bars of chocolate and half a liter of chocolate milk within about an hour. While eating, she feels like she can't stop and feels the urge to keep on eating. Afterwards, she often suffers from nausea and is angry with herself. She considers herself “weak and undisciplined”. After the described eating episodes, she does not engage in any immediate behavior to prevent weight gain, but she is already planning the next diet. She is sad that she is “so fat and ugly” and only wears loose clothing to hide her body. She avoids shopping for clothes with friends, which she used to enjoy doing.

*BED - non-stereotypical:* Ms. G., 27 years old, reports that she has “big problems with food” and “just doesn’t know what to do anymore.” Even at primary school age, she had a higher weight than most of her peers, which is why her mother “put her on a diet early on”. She currently weighs 81 kg and is 170 cm tall (BMI 28 kg/m²). Her eating habits have been “completely out of control” for some time now: for about 1.5 years now, she has been “shoving down an extremely large amount of food at once” about every two to three days, even though she is not hungry at all. In such situations, for example, she consumes two scoops of vanilla ice cream and five caramel candies within about an hour. While eating, she feels like she can't stop and feels the urge to keep on eating. Afterwards, she often suffers from nausea and is angry with herself. She considers herself “weak and undisciplined”. After the described eating episodes, she does not engage in any immediate behavior to prevent weight gain, but she is already planning the next diet. She is sad that she is “so fat and ugly” and only wears loose clothing to hide her body. She avoids shopping for clothes with friends, which she used to enjoy doing.

*BED - non-pathological:* Ms. G., 27 years old, reports that even at primary school age, she had a higher weight than most of her peers. She currently weighs 81 kg and is 170 cm tall (BMI 28 kg/m²). She enjoys good food, especially sweets. She especially enjoys “snacking” while watching TV. Every two to three days, after she has already eaten dinner, she eats, for example, two scoops of vanilla ice cream and five caramel candies within about an hour. Since she “just likes” the taste of such sweets, she doesn’t try to eat less of them. She stops eating when she “has had enough.” Although her mother has already commented critically about her weight several times, Ms. G. feels comfortable in her body. She doesn't want to go on a diet or lose weight. She likes to wear nice clothes and enjoys going shopping for new clothes with her friends.

*AN - stereotypical:* Ms. B., 25 years old, says she has always been slim, enjoyed exercising lot and watched her figure. About a year and a half ago, she lost about 10 kg within 6 months through a diet, which meant that she weighed about 50 kg at a height of 168 cm (BMI 17.7 kg/m²). She felt very comfortable with this and also received many compliments, which is why she continued the diet. By now, she severely restricts her food intake, usually to just one small meal a day, rarely two. She completely avoids carbohydrates and prepares meals with as little fat as possible. She now finds greasy food “really disgusting”. Even though she has continued to lose weight and now only weighs 43kg (BMI 15.2 kg/m²), she is very afraid of gaining weight back. She checks her reflection in the mirror several times a day and counts every calorie she consumes, which she finds strenuous but to her “it’s worth it”. She also goes for a run at least 5 times a week. She feels comfortable with her current weight, but her social life and her relationship are suffering severely, because she is no longer willing to go out to restaurants or to cook together. Because others have little understanding of her concerns about her weight, she has withdrawn a lot overall.

*AN - non-stereotypical:* Ms. B., 25 years old, says she has always been curvy, but enjoyed exercising lot and watched her figure. About a year and a half ago, she lost about 16 kg within 6 months through a diet, which meant that she weighed about 64 kg at a height of 168 cm (BMI 22.7 kg/m²). She felt very comfortable with this and also received many compliments, which is why she continued the diet. By now, she severely restricts her food intake, usually to just one small meal a day, rarely two. She completely avoids carbohydrates and prepares meals with as little fat as possible. She now finds greasy food “really disgusting”. Even though she has continued to lose weight and now only weighs 58kg (BMI 20.6 kg/m²), she is very afraid of gaining weight back. She checks her reflection in the mirror several times a day and counts every calorie she consumes, which she finds strenuous but to her “it’s worth it”. She also goes for a run at least 5 times a week. She feels comfortable with her current weight, but her social life and her relationship are suffering severely, because she is no longer willing to go out to restaurants or to cook together. Because others have little understanding of her concerns about her weight, she has withdrawn a lot overall.

*AN - non-pathological:* Ms. B., 25 years old, says she has always been slim, enjoyed exercising lot and watched her figure. About two years ago, she lost about 5 kg within 4 months through a diet, which meant that she weighed about 58 kg at a height of 168 cm (BMI 20.6 kg/m²). Since then, her weight has remained constant and she feels comfortable with her body. She only restricts her food once a year, during Christian Lent. She then completely avoids sweets and all foods with added sugar. During these weeks she usually loses around 1-2 kg, but later gains it back again. She goes for a run at least twice a week. Many people in their social environment are also fit and pay attention to a healthy, balanced diet. She has a large circle of friends and often meets up with friends to go out to restaurants or cook dinners together.

*BN - stereotypical:* Ms. M., 22 years old, reports that she has been having recurring “binge eating attacks” for about two years. These occur about twice a week, usually in the late afternoon and last for about one to two hours. During these episodes, she “completely loses control” over how much and what she eats. For example, she would eat half a package of cookies (100 g) and a cheese pizza. Afterwards, she feels as if she was “about to burst” and is “disgusted” with herself. Because she is very afraid of gaining weight, she wants to “get rid of the calories quickly.” She then goes into the bathroom and induces vomiting by sticking the end of her toothbrush or a finger down her throat. Afterwards she feels tired and very ashamed because she “again hadn’t managed to eat normally”. She still feels depressed in the days afterward. She is very dissatisfied with her body. Her current weight, which she checks daily and which has been largely constant for several years, is 59 kg at a height of 1.73 m (BMI 19.7 kg/m^2^). She wants to lose at least three kilos, so she pays a lot of attention to a “low-fat diet” and does strength training in the gym three to four times a week. She feels like a failure because she has not yet reached her goal weight because of the “binge eating attacks”.

*BN - non-stereotypical:* Ms. M., 22 years old, reports that she has been having recurring “binge eating attacks” for about two years. These occur about twice a week, usually in the late afternoon and last for about one to two hours. During these episodes, she “completely loses control” over how much and what she eats. For example, she would eat half a package of cookies (100 g) and a cheese pizza. Afterwards, she feels as if she was “about to burst” and is “disgusted” with herself. Because she is very afraid of gaining weight, she wants to “get rid of the calories quickly.” She then trains on her spinning bike for about an hour and a half, even when she is already exhausted. She skips other planned activities, such as meeting up with friends, in favor of exercise. Afterwards she feels tired and very ashamed because she “again hadn’t managed to eat normally”. She still feels depressed in the days afterward. She is very dissatisfied with her body. Her current weight, which she checks daily and which has been largely constant for several years, is 59 kg at a height of 1.73 m (BMI 19.7 kg/m^2^). She wants to lose at least three kilos, so she pays a lot of attention to a “low-fat diet” and does strength training in the gym three to four times a week. She feels like a failure because she has not yet reached her goal weight because of the “binge eating attacks”.

*BN - non-pathological:* Ms. M., 22 years old, reports that she has been race cycling as a hobby for about two years. During the week, she covers a distance of 50 km on three to four days each. At the weekend, she usually goes on a longer ride of up to 100 km. When the weather is bad, she trains on her spinning bike. If something comes up when a training session was planned, such as a spontaneous meeting with friends, she skips her training if necessary. Since she started cycling, she has noticed that she is hungrier and eats more compared to before. On training days, she consumes around 3,000 calories. Before training, she often eats 250 g of low-fat quark with 100 g of oat flakes and some honey, and after training another 250 g of low-fat quark with a few berries. Her friends are sometimes surprised at how much she eats, but it doesn't bother her. Her diet gives her “the energy she needs for cycling.” She currently weighs 59 kg at a height of 173 cm (BMI 19.7 kg/m²), her weight has been largely constant for several years. She feels comfortable in her body.

**Intervention (original intervention used within the study was in German; an English translation is provided below)**

*Intervention group:* The prevalence of eating disorders has increased over the last few years. Despite the high level of suffering and the possibility of serious physical damage, many of those affected receive treatment very late or no treatment at all. One of the reasons for this is that doctors and therapists often overlook the problem if the clinical presentation is not typical. Those affected who do not fit the stereotypical image of a person with an eating disorder often do not receive a diagnosis and no treatment, even though they meet the diagnostic criteria.

*Control group:* The prevalence of eating disorders has increased over the last few years. This means that diagnosing eating disorders is becoming increasingly relevant for doctors and therapists, as the number of affected individuals presenting in practices, outpatient clinics, or clinics is increasing. Doctors and therapists have to assess more frequently in their everyday work whether an eating disorder is present and whether treatment is needed.
